# P065 Diagnostic complexity of overlapping head and neck pathologies in giant cell arteritis

**DOI:** 10.1093/rap/rkag113.095

**Published:** 2026-11-03

**Authors:** Knev Mashida, Luke Williamson

**Affiliations:** Barts Health NHS Trust, London, United Kingdom; Barts Health NHS Trust, London, United Kingdom

## Abstract

**Introduction:**

New craniofacial and cervical symptoms in patients with giant cell arteritis may represent relapse, unrelated pathology or an interaction between several coexisting processes. Distinguishing these possibilities is difficult during glucocorticoid tapering, when delayed treatment risks ischaemic complications but unnecessary escalation carries cumulative harm. We describe a patient with biopsy-proven giant cell arteritis who developed recurrent head, facial, jaw and neck symptoms alongside neurological, cervical and structural head-and-neck pathology, raising questions about both diagnostic attribution and possible interrelationships between these presentations.

**Case description:**

A 75-year-old woman presented with severe headache and diplopia and was treated with prednisolone 60 mg daily. Right temporal artery biopsy demonstrated transmural arteritis with multinucleated giant cells, confirming giant cell arteritis. Her headache and diplopia resolved shortly after commencing prednisolone.

During tapering she developed soreness over the vertex and posterior neck, which she consistently described as different from her presenting headache. Increasing prednisolone to 20 mg daily produced no improvement, however had no recurrent diplopia or visual loss. Subsequent erythrocyte sedimentation rate was 5 mm/hour, C-reactive protein was below 1 mg/L and computed tomography venography was normal. Giant cell arteritis was considered to be in remission and a cervicogenic contribution was suspected.

Over the following year, persistent head and neck symptoms occurred in the context of pre-existing trigeminal neuralgia, cervical degenerative change on magnetic resonance imaging and an enlarging Warthin tumour. Following tumour resection, neck discomfort initially improved, although ipsilateral auricular numbness persisted postoperatively. Prednisolone was successfully discontinued in December 2025 without recurrence of her original symptom pattern.

At age 77, seven months after stopping prednisolone, she reported ongoing unilateral neck pain, diffuse scalp soreness and new persistent jaw and tooth pain. Erythrocyte sedimentation rate was 8 mm/hour. She denied her original headache, fatigue and visual symptoms. Examination showed unilateral cervical muscle tenderness without focal upper-limb neurological deficit.

Dental assessment identified no dental cause her symptoms were attributed to mandibular trigeminal neuralgia. The pain subsequently appeared responsive to nightly carbamazepine, with cervical facet joint injections planned for the ongoing cervicogenic neck pain.

**Discussion:**

This case demonstrates the difficulty of interpreting evolving head and neck symptoms in a patient with proven giant cell arteritis and several coexisting regional pathologies. The clinical problem was not limited to deciding whether each symptom represented relapse or an unrelated mimic, but also raised the possibility that inflammatory, neurological, musculoskeletal, structural and postoperative processes may have overlapped or influenced one another.

Several findings reduced the likelihood that active giant cell arteritis alone explained the later symptoms. The pain differed from the original severe headache, inflammatory markers remained low, visual symptoms and scalp tenderness were absent, and increasing prednisolone to 20 mg daily did not improve one symptomatic episode. Cervical degeneration, trigeminal neuralgia, a Warthin tumour and postoperative sensory impairment each provided plausible explanations for components of the symptom complex. Successful glucocorticoid withdrawal without recurrence of the original presentation further supported remission.

The patient’s language was particularly important when the new jaw symptoms developed. She described continuous jaw and tooth pain rather than progressive pain or fatigue brought on by chewing or prolonged talking, making classical jaw claudication less likely. Subsequent dental assessment excluded a dental cause and identified mandibular trigeminal neuralgia, with apparent improvement following carbamazepine. Meanwhile, the planned cervical facet joint injections reflected a separate mechanical explanation for the persistent neck pain.

These findings support multiple concurrent pain mechanisms rather than a single alternative diagnosis. However, they do not prove that the conditions were wholly independent. Cervical referred pain, trigeminal dysfunction, local tumour-related change and postoperative nerve impairment may have contributed separately or additively to symptom localisation.

The case highlights the value of precise symptom description, longitudinal comparison with the original vasculitic presentation and targeted multidisciplinary assessment. Continued safety-netting remains essential because normal inflammatory markers and credible alternative diagnoses do not independently exclude future relapse.

**Key learning points:**

• How a patient describes jaw pain can be central to distinguishing possible giant cell arteritis relapse from another cause. It is key to determine the exact nature of the pain in contrast to the typical vasculitic presentation of progressive discomfort or fatigue typically brought on by chewing or prolonged talking in jaw claudication.

• Comparison with the patient’s original giant cell arteritis presentation may be more informative than the location of pain alone. Differences in quality, triggers, associated symptoms and temporal pattern can meaningfully alter the likelihood of relapse.

• More than one process may contribute to symptoms at the same time. In this case, trigeminal neuralgia, cervical degenerative disease, postoperative sensory change and previous salivary gland pathology each offered a possible explanation for different aspects of the presentation.

• Targeted specialist assessment and response to treatment can help clarify which mechanism is most likely. The absence of a dental cause and improvement with carbamazepine supported mandibular trigeminal neuralgia, while planned facet joint injections reflected a separate cervical pain component.

• Normal inflammatory markers and credible alternative diagnoses reduce the likelihood of active vasculitis but do not remove it entirely. Ongoing review remains important if symptoms change, fail to respond as expected or develop features more suggestive of ischaemia, with a low threshold for further investigation of these symptoms.

